# Identification of a Neutralizing Monoclonal Antibody That Recognizes a Unique Epitope on Domain III of the Envelope Protein of Tembusu Virus

**DOI:** 10.3390/v12060647

**Published:** 2020-06-15

**Authors:** Shenghua Qu, Xiaoyan Wang, Lixin Yang, Junfeng Lv, Runze Meng, Weiqian Dai, Qingxiangzi Li, Huicong Liu, Bing Zhang, Dabing Zhang

**Affiliations:** Key Laboratory of Animal Epidemiology of the Ministry of Agriculture, College of Veterinary Medicine, China Agricultural University, Beijing 100193, China; qushenghua@cau.edu.cn (S.Q.); wxy320@cau.edu.cn (X.W.); yanglixin@cau.edu.cn (L.Y.); junfeng1017@cau.edu.cn (J.L.); b20173050386@cau.edu.cn (R.M.); wqdai@ips.ac.cn (W.D.); s20183050722@cau.edu.cn (Q.L.); liuhuicong@cau.edu.com (H.L.)

**Keywords:** duck, Tembusu virus, monoclonal antibody, neutralizing antibody, antibody-mediated neutralization, epitope

## Abstract

Domain III of the envelope protein (EDIII) is the major target of flavivirus neutralizing antibody. To date, little is known regarding antibody-mediated neutralization of Tembusu virus (TMUV), a novel flavivirus emerging in duck in 2010. Here, a novel monoclonal antibody (MAb), designated 12F11, was prepared by immunization of mice with recombinant EDIII (rEDIII) protein. Using virus neutralization test, 12F11 in undiluted ascites neutralized the TMUV infectivity to induce the development of cytopathic effects in BHK-21 cells, indicating that 12F11 exhibits a neutralizing activity. The neutralizing activity of 12F11 was confirmed by plaque reduction neutralization test, in which 12F11 reduced significantly the number of plaques produced by TMUV in BHK-21 cells. Western blot analyses of a series of truncated rEDIII proteins showed that the epitope recognized by 12F11 includes amino acids between residues 8 and 77 of EDIII protein. Function analysis demonstrated that 12F11 neutralizes TMUV infection at virus adsorption and at a step after adsorption to a certain extent. The present study provides an important step towards elucidating antibody-mediated neutralization of TMUV.

## 1. Introduction

Tembusu virus (TMUV), one of the members in the genus *Flavivirus*, was first isolated from mosquitoes in 1955 in Malaysia [1]. The virus caused explosive outbreaks of a severe disease in ducks in eastern China at the end of spring and the beginning of summer of 2010. In the next six months, the disease spread rapidly to other regions with intensive duck productions, including southern China, central China, and northern China [2,3,4]. It is estimated that about 120 million egg-laying and 15 million meat-type breeder ducks were affected in 2010, resulting in serious economic losses to the Chinese poultry industry. Multiple strategies have been employed for generating effective vaccines [5,6,7,8,9,10,11]. A recent study showed that TMUV mediates antibody-dependent disease severity in mice [12]. It is therefore crucial to define the epitopes which induce the potent neutralizing antibodies for development of a safe and effective TMUV vaccine.

As with other flaviviruses, TMUV has a positive-sense, single-stranded RNA genome, encoding a large polyprotein which was predicted to be cleaved into three structural proteins, capsid (C), precursor of membrane/membrane (prM/M), and envelope (E), and seven nonstructural proteins, NS1, NS2A, NS2B, NS3, NS4A, NS4B, and NS5 [4,13,14,15,16]. The flavivirus E protein is the major protein on the surface of virion. During cell infection, it plays an important role in virus entry into host cell, because it binds to receptors and mediates fusion of the viral membrane with endosomal membranes. Because of this, the E protein is the major target of flavivirus neutralizing antibodies. Binding of antibodies to E protein can result in direct block of receptor binding and inhibition of endocytosis and membrane fusion, which are considered important mechanisms of antibody-mediated neutralization [17,18,19]. The E protein ectodomain is composed of three structural domains, EDI, EDII, and EDIII [20,21]. Previous studies have shown that all the three domains contain epitopes eliciting neutralizing monoclonal antibodies (MAbs), whereas many of the most potent neutralizing MAbs are mainly elicited by epitopes located on the EDIII domain [17]. Several studies have shown that neutralizing antibodies are indicative of a long-term immunity acquired from infection and vaccination [22].

Some progress in immune protection against TMUV has been made in China. Firstly, the TMUV E protein was shown to induce protective immune response by different approaches, including immunization with E protein delivered by recombinant duck enteritis virus [5,9], and expressed in a prokaryotic system [7,10]. Furthermore, several mouse monoclonal antibodies (MAbs) (1F3, 1A5, 3B6, 3E9, 3H11, and 1G2) against E protein have been described [23,24,25,26,27]. Importantly, MAbs 3E9 and 1G2 exhibit neutralizing activity [24,27]. Finally, three epitopes, recognized by non-neutralizing MAbs 1F3, 1A5, and 3B6, have been identified in EDII (positions 87–91 and 221–225) and EDIII (positions 374–380) [25,26]. The epitope recognized by neutralizing MAb 1G2 were mapped to the hi-loop of EDII [27].

In this study, we generated and characterized a mouse MAb (12F11) against the EDIII protein of TMUV. The MAb was shown by virus-neutralization assay and plaque reduction neutralization test (PRNT) to exhibit neutralizing activity. To provide new information regarding antigenic structure and antibody-mediated neutralization of TMUV, the epitope recognized by 12F11 was identified by using Western blot analyses of a series of truncated recombinant EDIII (rEDIII) proteins and the EDIII protein on virion. Furthermore, the role of 12F11 in blocking TMUV infection was also investigated by using pre- and post-adsorption inhibition assays.

## 2. Materials and Methods 

### 2.1. Ethics Statement

The protocols involving animals were approved by the Animal Welfare and Ethics Committee of China Agricultural University (license number CAU20161215-2).

### 2.2. Cells, Virus and Antiserum

BHK-21 cells were maintained in Dulbecco’s modified Eagle’s medium (DMEM; Macgene, Beijing, China) supplemented with 10% fetal calf serum (FCS; GIBCO, California, USA), 100 U penicillin, and 0.1 mg/mL streptomycin. 

The TMUV Y strain was originally isolated in 2014 in China from 74-day-old ducks exhibiting paralysis typical of TMUV infection and propagated in 9-day-old embryonated Peking duck eggs. The virus was propagated in BHK-21 cells, and stored at −80 °C until use. Virus titer was determined by plaque assay in BHK-21 cells and is expressed as plaque forming unit (PFU).

Antiserum against TMUV was produced by inoculation of 1-week-old Peking ducks with strain Y at the dose of 2 × 10^4^ PFU per duck intramuscularly. Serum samples were collected from surviving ducks at 7 days post inoculation (p.i.).

### 2.3. Preparation of rEDIII Protein

The E sequence (GenBank Accession No. MK542820) of TMUV Y was employed to predict the boundaries of EDIII with the conserved domain database (CDD) search [28]. The secondary structure of the EDIII protein was predicted by alignment with those of Dengue virus (DENV; PDB Accession No. 2JSF), Japanese encephalitis virus (JEV; GenBank Accession No. ABC79599.1), and West Nile virus (WNV; PDB Accession No. 1S6N) with Clustal Omega (https://www.ebi.ac.uk/Tools/msa/clustalo/) as well as the ESPript 3.0 analysis (http://espript.ibcp.fr/ESPript/ESPript/). Molecular mass (M_r_) of the EDIII proteins was predicted with ProtParam (https://web.expasy.org/protparam/).

TMUV EDIII-encoding gene was amplified from TMUV Y by using reverse transcription (RT)-PCR with primer 1f and 109.1r (Table 1) and cloned into expressing vector PET-28a, generating recombinant vector PET-28a-EDIII. Subsequently, PET-28a-EDIII was transformed into *Escherichia coli* (*E. coli*) Rosetta (DE3). Protein expression was induced by addition of isopropyl β-D-thiogalactopyranoside (IPTG) in culture. Plasmid PET-28a was included in the experiment and served as a control. The expressed rEDIII protein, containing an N-terminal 6× His tag, was purified using a Ni-Agarose Resin (CWbiotech, Beijing, China), and confirmed by 15% sodium dodecylsulfate polyacrylamide gel electrophoresis (SDS-PAGE) analysis. The rEDIII protein was characterized further by Western blot with anti-His MAb (Biodragon, Beijing, China) as primary antibody and horseradish peroxidase (HRP)-conjugated goat anti-mouse IgG (Biodragon, Beijing, China) as secondary antibody. Anti-TMUV duck serum and HRP-conjugated goat anti-duck IgG (Biodragon, Beijing, China) were also used in characterization. Protein concentration was measured using a Protein Quantitative Kit (Transgen, Beijing, China). 

The purified proteins were separated by SDS-PAGE, followed by staining of the gel with Coomassie brilliant blue. After destaining with solution consisting of 10% acetic acid, 30% methanol and 60% water, the proteins were harvested respectively, and analyzed by using the mass spectrometry method. The analysis was performed in Biomass Spectrometry Laboratory, College of Biological Sciences, China Agricultural University.

### 2.4. MAb Preparation 

Six-week-old BALB/c female mice were subcutaneously immunized three times at 14-day intervals with rEDIII protein (50 μg/mouse) emulsified in Freund’s complete or incomplete adjuvant. The mice, which were shown by an indirect enzyme-linked immunosorbent assay (ELISA) to display serum antibody titers of higher than 1:10,000, received the fourth immunization with rEDIII protein (100 μg/mouse). Three days later, spleen cells were prepared from the mice and fused with SP2/0 cells according to the standard procedure [29]. Hybridoma cells secreting anti-EDIII specific MAbs were screened using ELISA. The hybridomas were subcloned three times with the limiting dilution method. Ascites was produced with multipara mice.

### 2.5. MAb Isotyping

The MAb isotyping was performed using an SBA Clonotyping System-HRP kit (Southern Biotech, Birmingham, USA), according to the manufacturer’s instructions.

### 2.6. Indirect ELISA

An indirect ELISA was used to screen hybridomas and to measure the titers of MAb existed in ascites, employing virus stock (2 × 10^5^ PFU/well) and purified His-tagged rEDIII protein (200 ng/well) as coat antigens, respectively. The ELISA protocol was similar to that described previously [30], except that the coated plates were blocked with 5% non-fat milk in phosphate-buffered saline (PBS) supplemented with 0.05% tween-20 and an HRP-conjugated goat anti-mouse IgG served as secondary antibody. Ascites was prepared in serial 10-fold dilutions with 5% non-fat milk.

### 2.7. Detection of Reactivity of MAb to EDIII

Western blot assay was conducted to detect the reactivity of MAb to rEDIII protein and virions that are present in supernatant prepared from BHK-21 cells infected with TMUV Y. In these analyses, crude extracts from *E. coli* expressing PET-28a vector and supernatant harvested from uninfected BHK-21 cells were included as controls. MAb and HRP-conjugated goat anti-mouse IgG (Biodragon, Beijing, China) served as the first and second antibody respectively. MAb and second antibody were prepared in 1500- and 4000-fold dilutions with 5% non-fat milk, respectively. 

### 2.8. Indirect Immunofluorescence (IIF) Assay

Confluent monolayers of BHK-21 cells grown in 24-well plates were inoculated with TMUV Y at a multiplicity of infection (MOI) corresponding to 0.01 PFU/cell. BHK-21 cells inoculated with an equal volume of maintenance medium consisting of DMEM supplemented with 2% FCS, 100 U/mL penicillin, and 0.1 mg/mL streptomycin were included as a control. Following adsorption at 37 °C for 1 h, cells were washed three times with PBS, and cultured with 500 μL of maintenance medium. Following incubation in a 5% CO_2_ atmosphere at 37 °C for 40 h, medium was removed, and the cells were washed three times with PBS. The cells were fixed with cold absolute alcohol for 20 min at room temperature. The ethanol was removed and the cells were washed three times. Each of the monolayers was inoculated with 200 μL of a 100-fold dilution of MAb-containing ascites diluted in PBS. After incubation at 37 °C for 1 h, the cells were washed three times, 5 min every time, and stained with 300 μL of an 80-fold dilution of fluorescein isothiocyanate (FITC)-conjugated goat anti-mouse IgG (Biodragon, Beijing, China). After further incubation at 37 °C for 1 h, the cells were washed again, and examined under fluorescence microscopy (Olympus, Tokyo, Japan).

### 2.9. Neutralization Assay

One hundred microliters of ascites, which were inactivated at 56 °C for 30 min, were mixed with an equal volume of TMUV Y (10^4^ PFU). The mixture was incubated at 37 °C for 1 h, and inoculated onto confluent monolayers of BHK-21 cells grown in 24-well plates. Following adsorption at 37 °C for 1 h, the inoculum was removed and the cells were washed three times with PBS. Five hundred microliters of maintenance medium were added, and incubation was continued for additional 3 days. The cells were examined daily for cytopathic effect (CPE). Each test included a virus control, which received a mixture consisting of 100 μL of maintenance medium and an equal volume of virus stock, and a negative control, which received 200 μL of maintenance medium.

BHK-21 cells at 36 h after inoculation with the TMUV Y plus 12F11 mixture in above experiment were subjected to IIF assay following the protocol as described above. To highlight cytoplasmic fluorescence, nuclei were stained at 37 °C for 1 h with 100 μL of 200-fold dilution of 4’, 6-diamidino-2-phenylindole (DAPI; Solarbio, Beijing, China).

### 2.10. PRNT

MAb 12F11 was purified from mouse ascites using a Protein G Spin Purification Kit (Transgen, Beijing, China). Purified 12F11 (1 mg/mL) was prepared in serial 5-fold dilutions with maintenance medium. One hundred microliters of MAb from each dilution were mixed with 100 µL of diluted virus (89 PFU, final virus concentration). The mixture was incubated at 37 °C for 1 h and inoculated onto BHK-21 cells. Following adsorption at 37 °C for 1 h, the inoculum was removed and the cells were washed three times with PBS. Five hundred microliters of overlay medium consisting of DMEM containing 2% low melting-point agarose (Macgene, Beijing, China) and 2% FCS were added. Following incubation in a 5% CO_2_ atmosphere at 37 °C for 3 days, the cells were fixed with 0.5 mL of 4% paraformaldehyde at room temperature for 90 min. Then, the paraformaldehyde and agarose were removed and the cells were stained with 0.5 mL of 0.2% (*w/v*) crystal violet. Fifteen minutes later, crystal violet was removed and plaques were read. This assay included a virus control, which received a mixture consisting of 100 μL of maintenance medium and 100 μL of virus stock, and a negative control, which received 200 μL of maintenance medium. The ability of MAb 12F11 to neutralize virus infectivity was measured with a neutralizing dose (ND_50_) as described previously [31].

### 2.11. Identification of the Epitope Recognized by 12F11

A series of truncated EDIII-encoding regions was amplified from recombinant plasmid PET-28a-EDIII using PCR with primers shown in Table 1, and cloned into vector PGEX-6P-1, generating a series of recombinant vectors (Table 1). Following transformation of the recombinant vectors into *E. coli*. Rosetta (DE3) and induction with IPTG, glutathione-S-transferase (GST)-fused truncated proteins was confirmed and separated by SDS-PAGE. The truncated rEDIII proteins with the expected sizes were purified using the gel extraction method, and characterized using Western blot assay with anti-GST MAb (Macgene, Beijing, China) as primary antibody and HRP-conjugated goat anti-mouse IgG as secondary antibody. The epitope recognized by MAb 12F11 was identified by detection of reactivity of the TMUV MAb to the truncated rEDIII proteins in Western blot assay.

### 2.12. Pre- and Post-Adsorption Inhibition Assays

Pre- and post-adsorption inhibition experiments were conducted as described previously [32]. Briefly, pre-chilled DMEM was used to dilute virus to give 40 PFU in 100 μL, and to dilute the purified MAb in serial 5-fold dilutions to 5^−5^. Confluent monolayers of BHK-21 cells were prepared in 24-well plates (2 × 10^5^/well) and washed three times with pre-chilled PBS until use. 

For the pre-adsorption analysis, 100 μL of MAb 12F11 from each dilution were mixed with 100 μL of virus, followed by incubation at 4 °C for 1 h. Then, each culture was inoculated with 200 μL of the TMUV Y plus 12F11 mixture. Virus adsorption was carried out at 4 °C for 1 h. For the post-adsorption analysis, each culture was inoculated with 100 μL of virus and 100 μL DMEM. Following adsorption at 4 °C for 1 h, pre-chilled PBS was used to washed out the unbound virus for three times. One hundred microliters of MAb from each dilution were mixed with 100 μL DMEM, and added onto cell cultures, and incubated at 4 °C for 1 h.

After the cells were washed three times with PBS, each culture was overlaid with 500 μL of overlay medium. Following incubation, fixation, and staining as described in PRNT, plaques were counted. The experiments were repeated for three times. In each experiment, a negative control was included, in which MAb was replaced with 100 μL of DMEM. Each experiment was repeated three times. The ability of MAb 12F11 to neutralize virus infectivity in pre-adsorption and post-adsorption inhibition experiments were measured by comparing the number of plaques formed in the MAb treatment group with the value obtained from the control group. The percentage of plaques formed in the presence of MAb (mean PFU obtained from the MAb treatment group/mean PFU obtained from the control group) was also calculated as reported previously [32].

### 2.13. Statistical Analysis

The data obtained in PRNT and pre- and post-adsorption inhibition experiments were calculated as mean ± standard deviation (SD). For comparison of groups, we used the t test implemented in the GraphPad Prism software version 6.01 (GraphPad Software Inc., San Diego, CA, USA). *p* < 0.05 was considered statistically significant.

## 3. Results

### 3.1. Expression and Characterization of the rEDIII Protein

**Figure 1:**
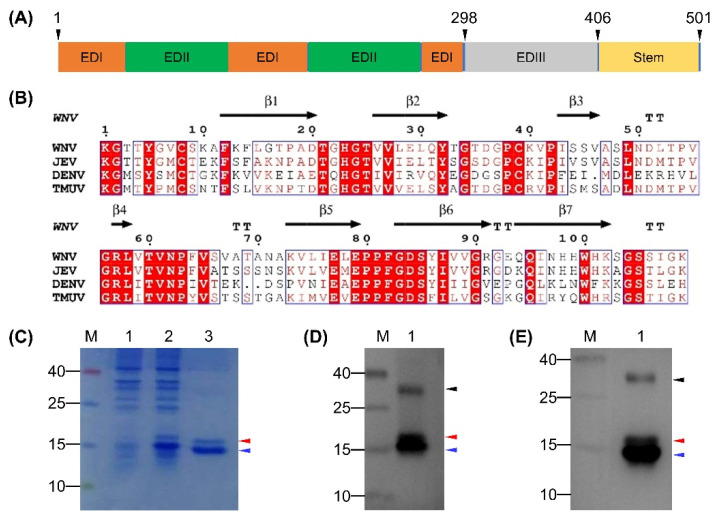
Prediction of domain III of the E protein for TMUV Y and characterization of rEDIII protein expressed in *E. coli*. (**A**) Diagram of E protein of TMUV Y. The predicted domains and stem are shown. The first and last residues of E protein and the positions of EDIII protein in the E protein are indicated above the gene box. (**B**) ESPript output from amino acid sequence alignment of EDIII protein of TMUV Y with those of DENV, JEV, and WNV. *β*-strands shown above the alignment refer to those of WNV. TT indicates strict β turns. (**C**) Analysis of rEDIII protein by SDS-PAGE. Lane M, molecular weight marker; Lane 1, crude extracts from *E. coli* before induction with IPTG; Lane 2, crude extracts from *E. coli* after induction with IPTG; Lane 3, purified rEDIII protein. (**D**) Analysis of rEDIII protein by Western blot using anti-His MAb. Lane M, molecular weight marker; Lane 1, purified rEDIII protein. (**E**) Analysis of rEDIII protein by Western blot using duck anti-TMUV serum. Lane M, molecular weight marker; Lane 1, purified rEDIII protein. The position of the expected 16.7-kDa rEDIII protein is indicated with red triangle. The positions of the unexpected 15.8- and 30-kDa proteins are indicated with blue and black triangles respectively. The numbers on the left of picture indicate molecular weight standards (in kDa).

The EDIII protein of TMUV Y was predicted to comprise 109 amino acids, which corresponded to residues 298–406 in the E protein (Figure 1A). The calculated M_r_ (11.7 kDa) was comparable to those of DENV (13.3 kDa), JEV (15.0 kDa), and WNV (12.2 kDa). Alignment of the EDIII protein of TMUV Y with those of DENV, JEV, and WNV whose three-dimensional structures are known revealed the detectable sequence conservation, which allowed a prediction of the amino acids making up seven β-strands (Figure 1B). On this basis, the rEDIII protein was expressed in *E. coli* Rosetta (DE3) cells. As shown in Figure 1C, a His-tagged rEDIII protein with the expected M_r_ of approximately 16.7 kDa was detected in both crude extracts after induction and purified products. Unexpectedly, an additional protein was detected, with M_r_ of approximately 15.8 kDa (Figure 1C). In the Western blot analysis, both 16.7- and 15.8-kDa proteins and an additional protein with M_r_ of approximately 30 kDa were recognized by anti-His MAb (Figure 1D) and duck anti-TMUV serum (Figure 1E). Using mass spectrometry analysis, the TMUV EDIII-specific amino acid sequences were detected in all three protein samples isolated from SDS-PAGE, with 109 amino acids (residues 1–109 in the EDIII protein) detecting in the 16.7-kDa protein, 101 amino acids (residues 2–102 in the EDIII protein) detecting in the 15.8-kDa protein, and 92 amino acids (residues 2–93 in the EDIII protein) detecting in the 30-kDa protein (Figure S1).

### 3.2. Preparation of MAb Directed Against TMUV EDIII

**Figure 2:**
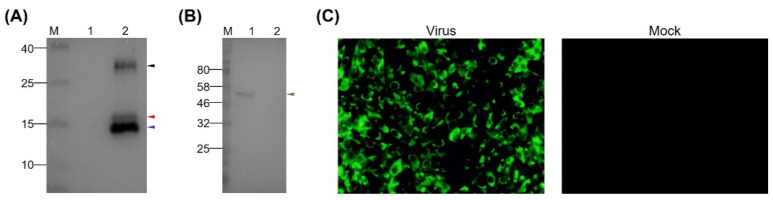
Identification of specificity of MAb 12F11. (**A**) Reactivity of 12F11 to rEDIII protein. Lane M, molecular weight marker; Lane 1, crude extracts from *E. coli* expressing PET-28a vector; Lane 2, purified rEDIII protein. (**B**) Reactivity of 12F11 to EDIII protein of virions. Lane M, molecular weight marker; Lane 1, virion-containing supernatant harvested from BHK-21 cells infected with TMUV Y; Lane 2, supernatant harvested from uninfected BHK-21 cells. The positions of the expected 16.7-kDa rEDIII protein and the 54-kDa E protein are indicated with red and green triangles respectively. The positions of the unexpected 15.8- and 30-kDa proteins are indicated with blue and black triangles, respectively. The numbers on the left of picture indicate molecular weight standards (in kDa). (**C**) Recognition of 12F11 to viral antigen expressed in infected cells using indirect immunofluorescence assay: (left) BHK-21 cells inoculated with TMUV Y and stained with 12F11 and FITC-conjugated goat anti-mouse IgG at 40 h post inoculation; and (right) uninfected BHK-21 cells.

To prepare MAbs recognizing the EDIII protein of TMUV, the purified rEDIII protein was employed to immunize six-week-old BALB/c female mice. A MAb, designated 12F11, was isolated after cell fusion as well as subcloning and screening of hybridoma cells. In Western blot analysis, 12F11 recognized all the three proteins described above (Figure 2A) and the EDIII protein on virion of TMUV Y (Figure 2B). In IIF test, BHK-21 cells infected with TMUV Y showed strong florescence signals (Figure 2C). No florescence was observed in uninfected BHK-21 cells (Figure 2C). Using an SBA Clonotyping System-HRP kit, 12F11 was identified to belong to subclass IgG1, with kappa (κ) light chain. The titer of antibodies in mouse ascites was determined to be approximately 1:4 × 10^6^ by ELISA using the purified rEDIII protein as coat antigen.

### 3.3. Analysis of Neutralizing Activity of MAb 12F11

**Figure 3:**
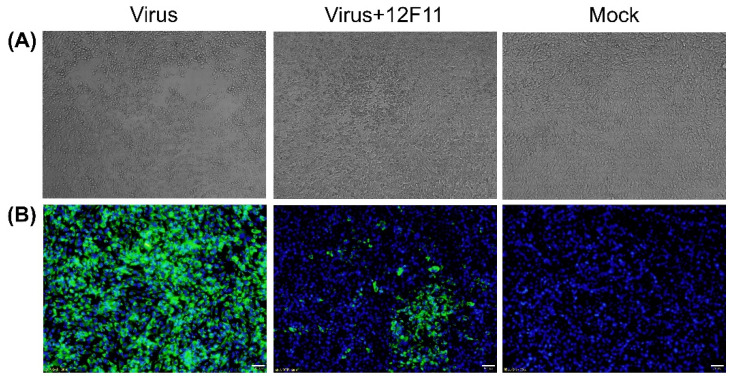
Investigation into 12F11-mediated neutralization. (**A**) Characterization of neutralizing activity of 12F11 using virus neutralization tests conducted in BHK-21 cells. The cells were observed for CPE at 70 h after inoculation with TMUV Y (Virus), TMUV Y plus 12F11 mixture (Virus + 12F11), and maintenance medium (Mock). Undiluted ascites was used in virus-antibody neutralization. (**B**) Assessment of neutralizing capacity of 12F11 using IIF assay. Inoculated BHK-21 cells in virus neutralization tests were sampled. The cells were stained with 12F11, FITC-conjugated goat anti-mouse IgG, and DAPI at 36 h after inoculation and viewed with a 20× magnification. Bar = 20 μm.

To investigate whether 12F11 possesses neutralizing activity, neutralization test was carried out in BHK-21 cells. As shown in Figure 3A, TMUV Y grew well, producing noticeable CPE within 48–72 h p.i. When the cells were incubated with mixture of TMUV Y and 12F11, no noticeable CPE was observed, whereas cell aggregations were seen in some areas. To investigate whether the cell aggregations were associated with virus infection, IIF analysis was conducted on the basis of neutralization test. As shown in Figure 3B, the infected culture showed strong green cytoplasmic florescence; the culture inoculated with the TMUV Y plus 12F11 mixture exhibited green florescence in specific areas; and only blue nuclei were seen in uninfected culture.

**Figure 4:**
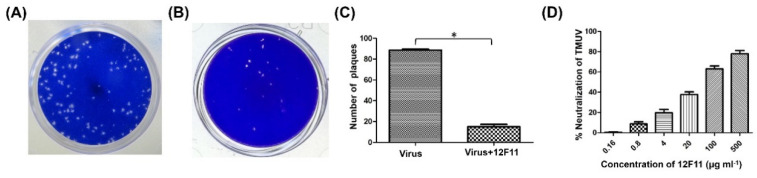
Evaluation of neutralizing potential of 12F11 using plaque reduction neutralization test. (**A**) Plaques formed in BHK-21 cells inoculated with TMUV Y. (**B**) Plaques formed in BHK-21 cells inoculated with TMUV Y plus 12F11 mixture. Undiluted 12F11 (500 μg/mL, final concentration) was used. (**C**) Number of plaques counted in BHK-21 cell cultures inoculated with TMUV Y and TMUV Y plus 12F11 mixture. The results shown are mean value ± SD of three independent experiments. * *p* < 0.05. (**D**) Percent neutralization of 12F11 with different concentrations. The results shown are mean value ± SD of three independent experiments.

PRNT was employed to evaluate further the neutralizing potential and to determine the neutralizing titer of purified 12F11. TMUV Y induced the formation of a large number of plaques in BHK-21 cells (Figure 4A). After incubation of TMUV Y with undiluted 12F11 (500 μg/mL, final concentration), only a small amount of plaques was produced by the virus (Figure 4B). In the three repeated tests, the number of plaques produced in the neutralization group was 15 ± 3, significantly lower than that (89 ± 1) in the virus control (*p* < 0.05) (Figure 4C). The ND_50_ titer of purified 12F11 was determined to be 13.8, corresponding to 36 μg/mL (Figure 4D).

### 3.4. Identification of Epitope Recognized by MAb 12F11 

**Figure 5:**
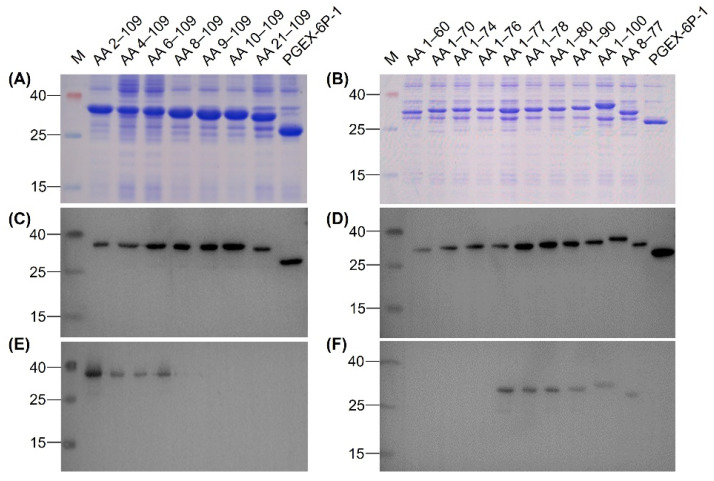
Identification of epitope recognized by 12F11. (**A**) Analysis of the N-terminal truncated rEDIII proteins by SDS-PAGE. (**B**) Analysis of the C-terminal truncated rEDIII proteins and the N- and C-terminal truncated rEDIII protein by SDS-PAGE. (**C**) Identification of the N-terminal truncated rEDIII proteins by Western blot assay using anti-GST MAb. (**D**) Identification of the C-terminal truncated rEDIII proteins and the N- and C-terminal truncated rEDIII protein by Western blot assay using anti-GST MAb. (**E**) Analysis of recognition of 12F11 to the N-terminal truncated rEDIII proteins. (**F**) Analysis of recognition of 12F11 to the C-terminal truncated rEDIII proteins and the N- and C-terminal truncated rEDIII protein. The truncated rEDIII proteins are shown above pictures. The numbers on the left of the pictures indicate molecular weight standards (in kDa).

To identify the epitope recognized by MAb 12F11, we expressed 17 N-terminal GST-fused truncated rEDIII proteins. All 17 truncated rEDIII proteins were expressed in *E. coli* successfully (Figure 5A,B), which were further confirmed by Western blot assay with anti-GST MAb (Figure 5C,D). 12F11 recognized the recombinant proteins with N-terminal truncations of 1–7 amino acids, including AA 2–109, AA 4–109, AA 6–109, and AA 8–109 (Figure 5E), and those with C-terminal truncations of 9–32 amino acids, including AA 1–100, AA 1–90, AA 1–80, AA 1–78, and AA 1–77 (Figure 5F). The MAb did not recognize the peptides with N-terminal truncations of 8–20 amino acids, including AA 9–109, AA 10–109 and AA 21–109 (Figure 5E), and those with C-terminal truncations of 33 to 49 amino acids, including AA 1–76, AA 1–74, AA 1–70, and AA 1–60 (Figure 5F). On this basis, we expressed the recombinant protein AA 8–77 in *E. coli* (Figure 5B,D). The recognition of the recombinant protein by 12F11 was confirmed by Western blot assay (Figure 5F).

### 3.5. Inhibitory Effect of MAb 12F11 on Virus Adsorption and a Step after Virus Adsorption

**Figure 6:**
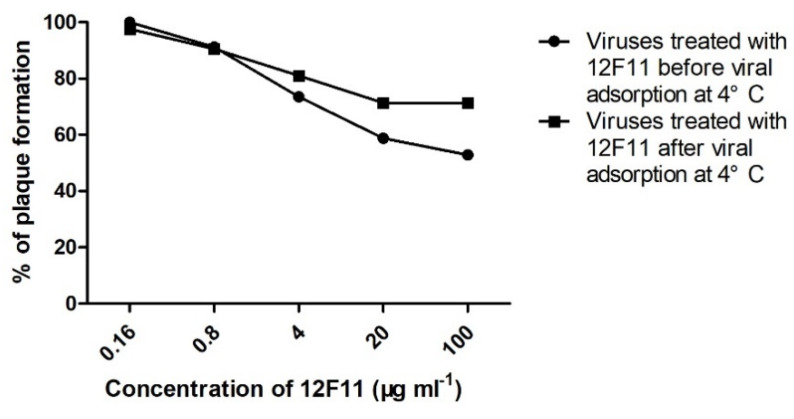
Neutralization of TMUV Y by treatment with MAb 12F11 in pre- and post-adsorption inhibition assays. TMUV Y was incubated with different dilutions of 12F11 before (●) or after (■) viral adsorption at 4 °C. The results shown are a percentage of plaque formation.

Pre- and post-adsorption inhibition assays were performed to investigate the ability of MAb 12F11 to neutralize TMUV infection before and after virus adsorption to host cells. In the pre-adsorption assay, numbers of plaques (18 ± 2 and 20 ± 7, respectively) formed by TMUV treated with 5- and 25-fold dilutions (corresponding to 100 and 20 μg/mL, respectively) of 12F11 were significantly lower than that (34 ± 5) of the control group (*p* < 0.05). The percentages of plaques formed in the presence of 5- and 25-fold dilutions of 12F11 were 52.9% and 58.8%, respectively (Figure 6). In the post-adsorption assay, numbers of plaques (30 ± 5, 30 ± 4, and 34 ± 2, respectively) formed by TMUV treated with 5-, 25-, and 125-fold dilutions (4 μg/mL) of 2F11 were significantly lower than that (42 ± 4) of the control group (*p* < 0.05). The percentages of plaques formed in the presence of 5-, 25-, and 125-fold dilutions of 12F11 ranged from 71.4% to 81% (Figure 6).

## 4. Discussion

In this paper, we describe the development of a novel mouse MAb targeting the EDIII protein of TMUV. On the basis of Western blot, indirect ELISA, and IIF, the 12F11 MAb was shown to recognized TMUV strain Y and to be specific for rEDIII of TMUV. Based on routine neutralization test and PRNT, we showed that 12F11 is a neutralizing MAb. We noted that BHK-21 cells inoculated with mixture of TMUV and 12F11, which was pre-incubated at 37 °C for 1 h, showed cell aggregations in some areas. Examination with IIF assay revealed florescence signals in specific areas, suggesting that TMUV cannot be neutralized completely by the MAb and still replicates in these areas. Compared with previously reported DENV-specific human MAb 5J7, which neutralizes 50% of virus at nanogram-range antibody concentration and is exceptionally potent [33], TMUV-specific mouse MAb 12F11 had a lower level of ND_50_ titer (36 μg/mL). Therefore, cell aggregations in cultures inoculated with the mixture of TMUV and 12F11 might be attributed to the lower titer of 12F11.

To date, two MAbs (1G2 and 3E9) exhibiting neutralizing activity antibodies have been reported for TMUV [24,27]. Of the two MAbs, 1G2 recognizes the hi-loop of EDII [27], whereas the epitope recognized by 3E9 has not been identified [24]. Thus, the epitope identified in this study may represent a novel neutralizing epitope. Western blot analyses of a series of truncated rEDIII proteins showed that residues Cys^8^ and Glu^77^ of the EDIII protein are crucial for 12F11 recognition, indicating the epitope recognized by 12F11 includes a long amino acid sequence between residues 8 and 77 of EDIII protein. It is not surprising that epitopes span a long sequence [34]. For example, seven MAbs (3H5, M8051122, 9F16, 2Q1899, 1P-05-143, GTX77558, and 5C36) specific to EDIII of DENV-2 recognize overlapping epitopes with residues Lys^305^ and Pro^384^ critical for binding [34]. Further studies are needed to determine whether the sequence represents a conformational epitope and to identify the critical antigenic sites in the epitope. 

The detection of the 15.8- and 30-kDa proteins in the purified products was unexpected. Nevertheless, the two proteins and the expected 16.7-kDa protein were all recognized by anti-His MAb, anti-EDIII MAb, and anti-TMUV serum, demonstrating that all the three proteins are specific for TMUV. Mass spectrometry analyses of the protein samples identified the TMUV EDIII-specific amino acid sequences, all of which include the epitope sequence between residues 8 and 77 of EDIII protein. Thus, the presence of the 15.8-, 16.7-, and 30-kDa proteins in the expressed product had no effect on preparation of 12F11. The recognition of the N-terminal His-tagged 15.8-kDa protein by anti-His MAb suggested that the protein might be a C-terminal truncated from of the 16.7-kDa protein. The present observation resembles previous findings of Gromowski and Barrett (2007), who reported that a truncated form appeared in expressing the DENV EDIII protein [34]. The formation of the 30-kDa protein remains to be clarified. We noted that the 30-kDa protein was almost invisible in SDS-PAGE analysis, whereas it was clear and distinguishable in the Western blot assay, which may be attributed to the lower quantity of the 30-kDa protein in purified products and high sensitivity of Western blot [35,36].

EDIII protein is the major target of flavivirus neutralizing antibody. The mechanisms of antibody-mediated neutralization include direct block of receptor binding and inhibition of internalization and membrane fusion [17,18,19]. In the investigation of mechanisms of MAb 12F11-mediated neutralization, preincubation of 12F11 with virus before viral adsorption greatly reduced the viral plaque formation at 20–100 μg/mL MAb concentrations. This suggests that 12F11 can neutralize TMUV infection at virus adsorption. Treatment of TMUV with 12F11 after viral adsorption also reduced formation of 19–28.6% plaques at 4–100 μg/mL MAb concentrations, suggesting that 12F11 can also neutralize TMUV infectivity to a certain extent at a step after viral adsorption to host cells.

Taken together, we have generated a mouse MAb 12F11 that recognizes a novel epitope within domain III of the E protein of TMUV. In vitro analysis revealed that the MAb exhibits neutralizing activity. Western blot analyses of a series of truncated rEDIII proteins showed that the epitope recognized by 12F11 includes amino acids between residues 8 and 77 of EDIII protein. Function analysis demonstrated that 12F11 neutralizes TMUV infection by blocking virus adsorption, and, to a certain extent, at a step after adsorption. The present study provides an important step towards elucidating antibody-mediated neutralization of TMUV.

## Figures and Tables

**Table 1:** Primers used for construction of recombinant plasmids expressing full-length EDIII protein and a series of truncated EDIII proteins.

Primer	Sequence (5′‒3′) ^d^	Recombinant Plasmid ^e^	M_r_ of Recombinant Protein (kDa) ^f^
1f	CGCGGATCCAAAGGAATGACCTACCCGATGTGTAGC (BamH I)	/	/
109.1r ^a^	CCGCCTCGAGTTTTTCCAATTGTGCTCCCACTTCTATG	PET-28a-EDIII	16.7
100r ^b^	CCGCTCGAGCCACTGGTACCTGATCTGTC (Xho I)	PGEX-6P-1(AA1‒100)	38.7
90r ^b^	CCGCTCGAGCCCTACTAAGATGAATGAATCCCCGAATGG	PGEX-6P-1(AA1‒90)	37.5
80r ^b^	CCGCTCGAGAGGTTCCACTTCCACCATTATCTTG	PGEX-6P-1(AA1‒80)	36.4
70r ^b^	CCGCTCGAGCGTGGAGGAGGTCGACACGTATGGGTTGAC	PGEX-6P-1(AA1‒70)	35.4
60r ^b^	CCGCTCGAGTTGTTATCAAGCGTCCAACTGGTGTC	PGEX-6P-1(AA1‒60)	34.3
78r ^b^	CCGCTCGAGCACTTCCACCATTATCTTGGCAC	PGEX-6P-1(AA1‒78)	36.2
77r ^b c^	CCGCTCGAGTTCCACCATTATCTTGGCACCCGTGGAG	PGEX-6P-1(AA1‒77)	36.1
76r ^b^	CCGCTCGAGCACCATTATCTTGGCACCCGTG	PGEX-6P-1(AA1‒76)	36.0
74r ^b^	CCGCTCGAGTATCTTGGCACCCGTGGAGGAGGTC	PGEX-6P-1(AA1‒74)	35.8
2f ^c^	CGCGGATCCGGAATGACCTACCCGATGTGTAGC	PGEX-6P-1(AA2‒109)	39.5
4f ^c^	CGCGGATCCACCTACCCGATGTGTAGCAACAC	PGEX-6P-1(AA4‒109)	39.3
6f ^c^	CGCGGATCCCCGATGTGTAGCAACACATTTTCC	PGEX-6P-1(AA6‒109)	39.0
8f ^c^	CGCGGATCCTGTAGCAACACATTTTCCCTAGTG	PGEX-6P-1(AA8‒109)	38.8
9f ^c^	CGCGGATCCAGCAACACATTTTCCCTAGTGAAG	PGEX-6P-1(AA9‒109)	38.7
10f ^c^	CGCGGATCCAACACATTTTCCCTAGTG	PGEX-6P-1(AA10‒109)	38.6
21f ^c^	CGCGGATCCACTGGGCATGGCACTGTCGTGG	PGEX-6P-1(AA21‒109)	37.4
109.2r	CCGCCTCGAGTTTTCCAATTGTGCTCCCACTTCTATG	/	/

^a^ Reverse primer paired with forward primer 1f to amplify the full-length EDIII-encoding region. ^b^ Reverse primers paired with forward primer 1f to amplify the EDIII-encoding regions with 27–147-nt truncations at 3′-terminal part, respectively. ^c^ Forward primers paired with reverse primer 109.2r to amplify the EDIII-encoding regions with 3–60-nt truncations at 5′-terminal part. Forward primer 8f was also paired with reverse primer 77r to amplify an EDIII-encoding region with a 21-nt truncation at 5′-terminal part and a 96-nt truncation at 3′-terminal part, which was used to construct recombinant plasmid PGEX-6P-1(AA8‒77). ^d^ Restriction enzyme sites are underlined and indicated in parenthesis. ^e^ AA, amino acid. Numerals in parenthesis indicate amino acid positions in EDIII protein of the Y isolate. ^f^ The 16.7-kDa EDIII protein included a 5-kDa peptide expressed by PET-28a vector itself. Other recombinant proteins included a 28-kDa peptide expressed by PGEX-6P-1 vector itself.

## Supplementary Materials

The following are available online at https://www.mdpi.com/1999-4915/12/6/647/s1. Figure S1: Characterization of rEDIII proteins by using mass spectrometry.
