# Supplementary material for: Identification of a Neutralizing Monoclonal Antibody That Recognizes a Unique Epitope on Domain III of the Envelope Protein of Tembusu Virus
Source: Viruses. 2020 Jun 15;12(6):647. doi: 10.3390/v12060647 (PMC7354527; doi:10.3390/v12060647)
Supplement: Supplementary file 1 [file viruses-12-00647-s001.pdf]

Supplementary Figure

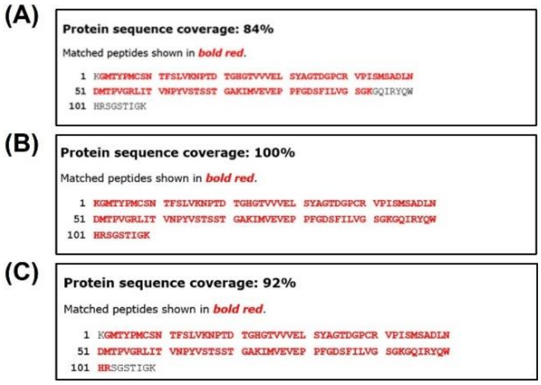

Figure S1. Characterization of rEDIII proteins by using mass spectrometry. (**A**) The 30 kDa protein. (**B**) The 16.7 kDa protein. (**C**) The 15.8 kDa protein.
